# Semen proteomics reveals alterations in fertility-related proteins post-recovery from COVID-19

**DOI:** 10.3389/fphys.2023.1212959

**Published:** 2023-11-09

**Authors:** Ankita Dash, Akanksha Salkar, Mehar Un Nissa, Prashant Makwana, Arundhati Athalye, Swapneil Parikh, Sanjeeva Srivastava, Firuza Parikh

**Affiliations:** ^1^ Department of Biosciences and Bioengineering, Indian Institute of Technology Bombay, Mumbai, India; ^2^ Department of Assisted Reproduction and Genetics, Jaslok-FertilTree International Centre, Jaslok Hospital and Research Centre, Mumbai, India

**Keywords:** infertility, mass spectrometry, post-recovery, reproductive health, SARS-CoV-2, semen proteomics

## Abstract

**Introduction:** Changes to sperm quality and decline in reproductive function have been reported in COVID-19-recovered males. Further, the emergence of SARS-CoV-2 variants has caused the resurgences of COVID-19 cases globally during the last 2 years. These variants show increased infectivity and transmission along with immune escape mechanisms, which threaten the already burdened healthcare system. However, whether COVID-19 variants induce an effect on the male reproductive system even after recovery remains elusive.

**Methods:** We used mass-spectrometry-based proteomics approaches to understand the post-COVID-19 effect on reproductive health in men using semen samples post-recovery from COVID-19. The samples were collected between late 2020 (1st wave, n = 20), and early-to-mid 2021 (2nd wave, n = 21); control samples were included (n = 10). During the 1st wave alpha variant was prevalent in India, whereas the delta variant dominated the second wave.

**Results:** On comparing the COVID-19-recovered patients from the two waves with control samples, using one-way ANOVA, we identified 69 significantly dysregulated proteins among the three groups. Indeed, this was also reflected by the changes in sperm count, morphology, and motility of the COVID-19- recovered patients. In addition, the pathway enrichment analysis showed that the regulated exocytosis, neutrophil degranulation, antibacterial immune response, spermatogenesis, spermatid development, regulation of extracellular matrix organization, regulation of peptidase activity, and regulations of calcium ion transport were significantly dysregulated. These pathways directly or indirectly affect sperm parameters and function. Our study provides a comprehensive landscape of expression trends of semen proteins related to male fertility in men recovering from COVID-19.

**Discussion:** Our study suggests that the effect of COVID-19 on the male reproductive system persists even after recovery from COVID-19. In addition, these post-COVID-19 complications persist irrespective of the prevalent variants or vaccination status.

## 1 Introduction

The severe acute respiratory syndrome coronavirus 2 (SARS-CoV-2) driven coronavirus disease 2019 (COVID-19) pandemic has greatly affected healthcare structures globally. With 616 million cases reported till October 2022 (WHO dashboard, 2022), the pandemic has also forced the research and medical community to work in collaboration to understand the virus. As the COVID-19 pandemic progressed, it brought to the fore the extra-pulmonary effects of COVID-19. Although discussed widely, the exact mechanism of the post-COVID-19 sequelae remains elusive. These extrapulmonary effects of COVID-19 are supported by two schools of thought 1) the effects are results of direct interaction with the virus and 2) indirect effects due to the systemic inflammation or other factors such as drugs given during the infection (Nie et al., 2021). Most of the direct interaction of the virus with organs other than the lungs is attributed to the fact that angiotensin-converting enzyme 2 (ACE2) and transmembrane protease, serine 2 (TMPRSS2) expression, which are critical for viral entry into the cell, are expressed in many other organs of the body (Dong et al., 2020). Interestingly, the expression of ACE2 and TMPRSS2 is very high in the testes. Also, reportedly males have a higher susceptibility to testing positive for COVID-19 (Yadav et al., 2021). Additionally, some studies have reported changes in the sperm and semen parameters post-COVID-19 (Ardestani Zadeh and Arab, 2021; Maleki and Tartibian, 2021; Mintziori et al., 2022). Therefore, studying the effect of COVID-19 on male reproductive health becomes crucial.

We designed a study to evaluate the implication of effects of COVID-19 on male reproductive health and infertility that persists after recovery. Our previously published study (Ghosh et al., 2022) was a pilot study to understand the post-COVID-19 effect on the male reproductive system. In this study, the semen samples were collected from patients who have recovered from COVID-19 and compared the alterations in the proteome with that of healthy individuals with no history of COVID-19. The study for the first time provided a comprehensive overview of alteration in whole semen proteome. Human semen constitutes secretions from the testis, epididymis, and male accessory glands such as seminal vesicles, prostate, and Cowper’s gland. The change in sperm formation, motility, and shape was attributed to alterations in proteins like semenogelin 1(SEMG1), cluster of differentiation 59 (CD59), prosaposin (PSAP), zona pellucida binding protein (ZPBP), sperm equatorial segment protein 1 (SPESP1), Dipeptidase 3 (DPEP3), sperm surface protein (SPA17), Outer dense fiber protein 2 (ODF2), and Neuropilin 1 (NRP1). These proteins are mapped to different pathways associated with spermatogenesis, motility, and fertilization. The study thus showed that the COVID-19 complications post recovery transcend beyond the respiratory complications. In addition, it emphasized the need to study the implication of COVID-19 on male reproductive health (Ghosh et al., 2022).

However, there have been incidences of COVID-19 resurgence driven by emerging SARS-CoV-2 strains, which have altered infectivity. This resurgence of COVID-19 has been popularly termed as “waves”. Different countries have reported such waves at different times (Wei et al., 2022). In India, apart from the initial wave from March to September 2020, driven by wildtype phenotype, there have been two incidences of resurgences driven by the delta and omicron variants (Kunal and Aditi, 2021). The delta variant was identified in 62% of the samples in April and 94% in May. This was also parallel to the advent of the second wave in India (Chakraborti et al., 2023). Delta variant has so far been reported to increase infectivity due to the mutations, although a few studies so far have reported changes in the pathogenesis of the virus (Harvey et al., 2021). The implications of these emerging variants on post-COVID-19 extrapulmonary complications also remain elusive. Another important thing that might have an impact on COVID-19 complications is the vaccine. Although vaccines can protect against variants or from infection, the exact effect of these vaccines on reducing the post-COVID-19 sequelae remains elusive (Taquet et al., 2022). Especially in India, where the vaccination campaign has received a huge response but with some setbacks due to misconceptions regarding the side effects of vaccines (Baig et al., 2022; Bansal et al., 2022). Therefore, understanding the effect of the variants and vaccines post-COVID-19 complications becomes crucial.

Post the initial COVID-19 wave, India witnessed another COVID-19 wave driven by the delta variant in April and May 2021. The delta was highly prevalent during the duration of sampling. Therefore, we believe that the patients from the second wave were prominently infected with the delta variant. As this variant was prevalent during the time of cohort recruitment and sample collection. In addition, India had also started the vaccination campaign by January 2021. Therefore, to understand the effect of variants and vaccines on the male reproductive system we collected semen samples from recovered patients infected during the two waves of COVID-19 in India.

## 2 Methods

### 2.1 Selection of participants for the study

The study was approved by the Institutional Review Board of Jaslok Hospital (vide letter no. EC/10509/2020 dated 12 November 2020). The patients were recruited for the study on receiving written consent. Semen samples were collected from 10 healthy individuals (control group) and 20 COVID-19-recovered individuals between September and December 2020 (1^st^ wave) and 21 samples between April and May 2021 (2^nd^ wave). The control group included fertile men who were seronegative for the antibody test against the SARS-CoV-2 antigen. The COVID-19-recovered group included patients who had earlier tested positive for SARS-CoV-2 by qRT-PCR by oral and nasopharyngeal swabs. The patients had mild-to-moderate symptoms during their hospitalization and were not on any antiviral therapy or steroidal prescriptions. Sample collection from the recovered patients was done after a minimum of 15 days post-recovery from COVID-19. Control individuals had typical semen parameters as per the WHO guidelines of 2010. The detailed clinical information is tabulated in Supplementary Table S1.

### 2.2 Inclusion and exclusion criteria

The COVID-19 recovered males that were recruited in the study were aged between 20 and 55 years of age. All of them had fathered at least one child by natural conception, and they had no history of infertility. All individuals enrolled in the study were non-diabetic, non-smokers, and did not consume alcohol. We ensured that the men included in the study did not have any history of prior exposure to any harmful chemical, radiation, or trauma that may otherwise be detrimental to their reproductive organs. Moreover, men with a demonstrated history of any abnormality such as azoospermia, asthenozoospermia, asthenoteratozoospermia, oligozoospermia, leukocytospermia, oligoasthenozoospermia, and oligoasthenoteratozoospermia were excluded. Further, those under supportive medication, such as steroids, chemotherapy, antiviral treatment, or other medications affecting the reproductive system, were excluded.

### 2.3 Sample collection, preparation, and processing for proteomics analysis

**FIGURE 1:**
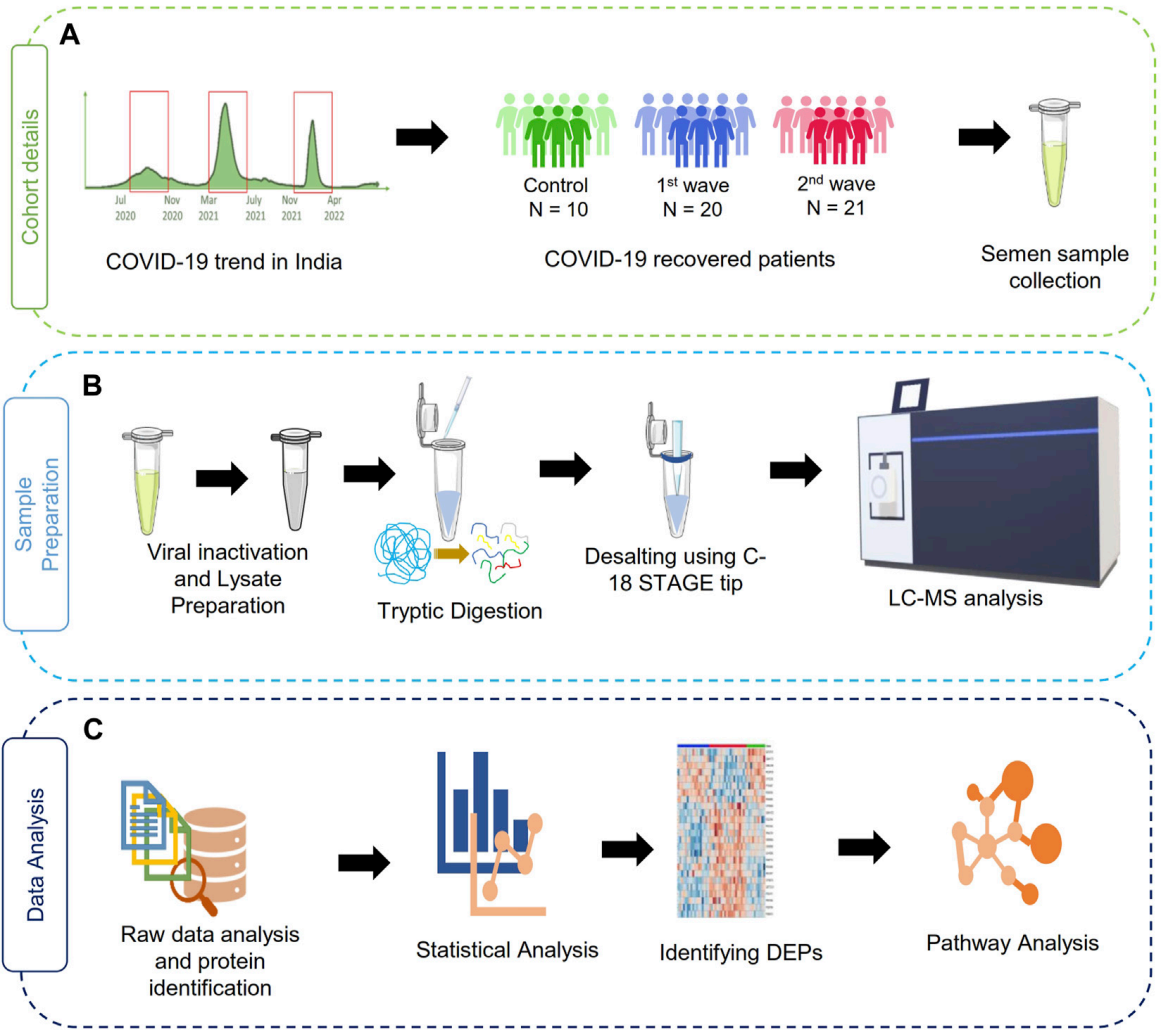
Schematic representation of the study design. **(A)** summary of a cohort of samples used in the study**, (B)** sample processing workflow for MS analysis, **(C)** data analysis and visualization pipelines for the discovery of proteomics data.

The sample collection, storage, assessment of semen parameters, and protein extraction were performed following the applicable guidelines and regulations of the WHO laboratory manual (2010). After 3 days of abstinence from any sexual activity, the semen samples were obtained by masturbation into sterile containers. After collection, the samples were kept at 37°C for 30 min for liquefaction before analysis. A dedicated room and laminar flow were assigned for this purpose. Semen parameters such as viscosity, pH, and volume were measured. Further sperm morphology was assessed using Qwik Check Diff Quik and a phase contrast microscope was used to determine sperm motility (Carl Zeiss Trinocular Microscope). Then, the samples were subjected to heat inactivation of the virus at 56°C for 30 min as a precautionary step before processing the samples for proteomics and then stored at −80°C. Further, the protein precipitation and lysate preparation were done as described by Ghosh et al., 2022. The study workflow has been illustrated in Figure 1.

The protein extracts were sent for proteomics analysis at MASSFIITB (Mass Spectrometry Facility at IIT Bombay). LFQ-based discovery proteomics was employed to investigate the persistent effect of viral infection on the male reproductive system using the protocol described by Ghosh et al., 2022. Further, 30 μg of the protein was taken forward for digestion after reduction and alkylation using Tris carboxyethyl phosphine and iodoacetamide, respectively. Samples were then subjected to overnight digestion using Trypsin (0.25 ug/ul) in a ratio of 1:30. The digested peptides were then desalted using a C-18 STAGE tip. The final peptides were reconstituted in Milli-Q water with 0.1% formic acid and quantified before submission for MS run. Finally, 1 μg of the peptide was injected for the LC–MS/MS run. A high-resolution Orbitrap Fusion Tribrid Mass Spectrometer coupled to an easy nano-liquid chromatography (LC) system was used in this study to acquire the proteomic data in a data-dependent manner. The settings used for the LC–MS/MS runs were as published earlier (Ghosh et al., 2022).

### 2.4 Data acquisition from the public repository

The raw files for the samples included in the previous study by Ghosh et al., 2022 were downloaded from the PRIDE repository (Dataset identifier: PXD026703). The files were then used to reanalyze the files using MaxQuant.

### 2.5 Quantitative proteomics analysis by MaxQuant

The mass spectrometric raw data sets were processed using the LFQ-based parameter in MaxQuant (version 1.6.6.0) (Tyanova et al., 2016) using the default parameters against the UniProt human database (downloaded on 11 March 2022). Furthermore, using the built-in search engine, Andromeda. The false discovery rate cutoff was set to 1% and proteins with more than 1 unique peptide were selected to increase the reliability of the data obtained. The detailed parameters of MaxQuant are given in Supplementary Figure S1.

### 2.6 Statistical analysis using MetaboAnalyst

The MaxQuant-analyzed files of 51 samples were taken forward for sample-wise correlation analysis on MetaboAnalyst (version 5.0) (Xia et al., 2009). The missing values of the features having LFQ intensities in more than 70% of each group were imputed separately by the KNN algorithm and considered for differential protein expression analyses. The LFQ intensities were log-transformed (base 10) and the median normalized data were taken for statistical analysis (i.e., *t*-test and fold change). One-way ANOVA was used for the comparison between the two waves and control samples. The posthoc analysis was done using Tukey’s HSD. The *p*-value threshold for the ANOVA and *post hoc* analysis was 0.05 and that for FDR was 0.1. Further individual comparisons between second wave vs. control samples and second wave vs. first wave were done using a *t*-test (Welch *t*-test) to identify the significant differential abundant proteins, where a *p*-value of 0.05 was set as the maximum threshold value. No FDR threshold was set for *t*-test. Among all the *t*-test passed proteins, proteins with fold change values greater than or equal to 1.5 were considered DEPs. GraphPad Prism 9 was used to visualize the box plot and heat maps.

### 2.7 Pathway and protein-protein interaction enrichment analysis

The biological process and pathway enrichment analysis were done in Metascape (version 3.5) (Zhou et al., 2019). The proteins identified to be significantly dysregulated on ANOVA test were used for pathway enrichment. Whole theoretical proteome was used as background proteome. In the pathway enrichment analysis, features having a *p*-value less than 0.01 and an enrichment score >1.5 were considered statistically significant. We also performed a pathway analysis comparing pathway enriched in 1^st^ and 2^nd^ wave using the *t*-test and fold-change threshold-passed proteins.

## 3 Results

The study included recovered patients from two waves of COVID-19 in India. Accordingly, the samples collected in late 2020 and mid-2021 were compared using a high-throughput proteomics approach to assess the overall alteration in semen proteome. Supplementary Table S1 summarizes the cohort characteristics. In addition, the study also included comparisons with control samples, i.e., samples from healthy individuals without any history of infertility or COVID-19 positivity. Compared to the control participants, there was a decrease in sperm parameters such as motility, morphology and count in COVID-19 recovered men even after around 30 days.

### 3.1 Label-free quantitative proteomics

Discovery-based proteomics data were obtained using high-resolution mass spectrometry analysis for 51 samples. Label-free quantification technique was used to quantify the semen proteome. The raw data for control and 1^st^ wave patients were previously published (Ghosh et al., 2022) and were downloaded and re-analyzed with that generated for the second-wave patients.

An average of **560** proteins were identified in each sample (Supplementary Figure S1A). After filtering proteins with more than 30% missing values, 381 proteins were used for further analysis. Missing values were imputed using the k-NN method. The imputed data was then normalized and log-transformed to make different comparisons. Since the samples were run at different times, to assess the effect of variations in instrument response, the data was subject to principal component analysis (PCA) to look at the potential batch effect. However, in the PCA plot, all the samples clustered together clearly indicating no batch effect; except for one outlier (SCT 33) (Supplementary Figure S1B). Some samples with low correlation were removed from further analysis (SCT1, SCT33, SCT41, and SCT52). These samples were also identified as outliers based on the PCA plot. We also observed overlap between the sample groups on PCA (Supplementary Figure S1C).

**FIGURE 2:**
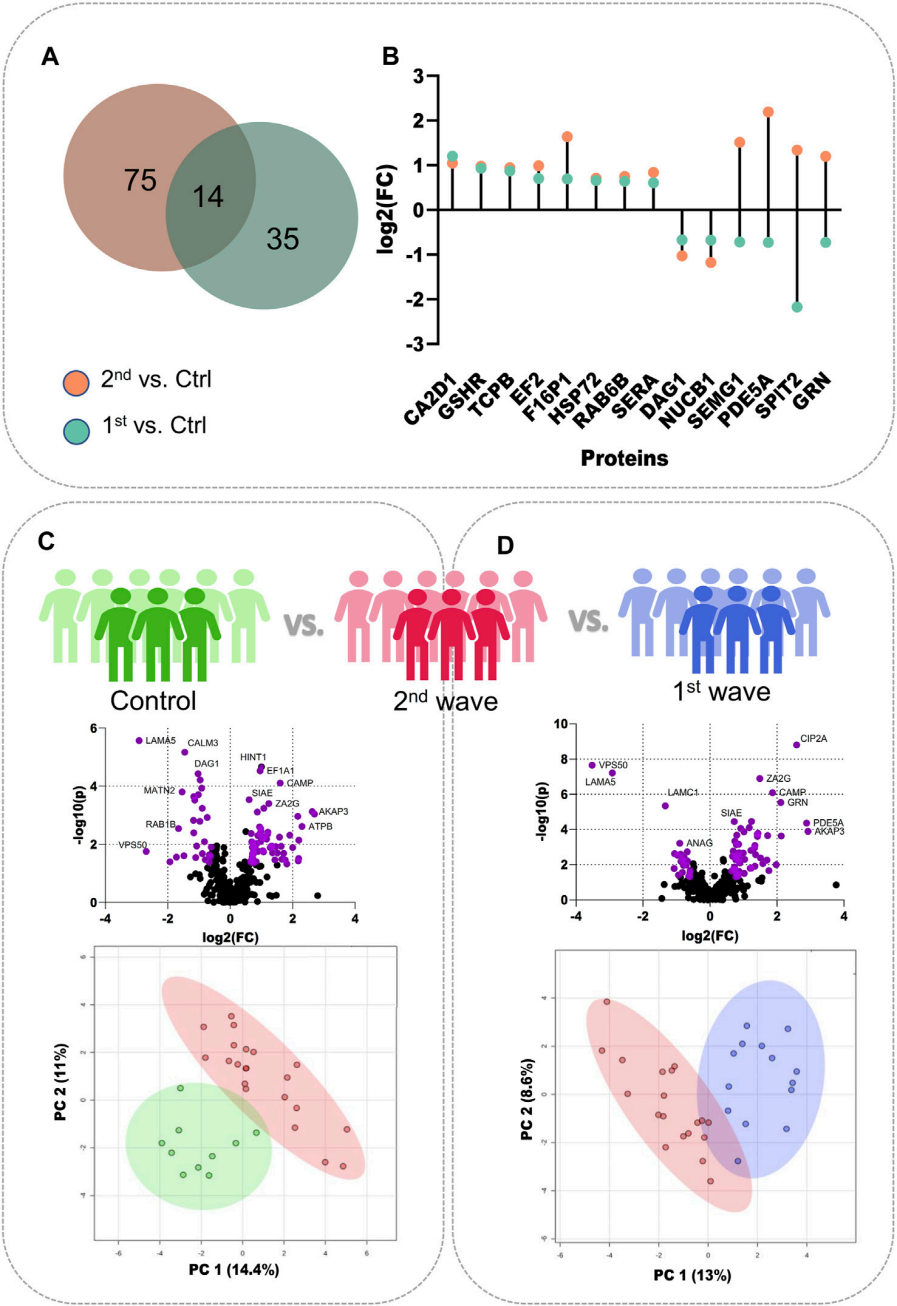
Individual comparisons between the different sample sets. **(A)** Venn diagram showing DEPs common and unique to the samples from two waves. **(B)** Dot plot showing the trends of 14 common DEPs identified from the two COVID-19 waves. **(C)** Volcano plot showing DEPs identified on comparing the control samples with 2^nd^ wave and the clustering on PCA, **(D)** Volcano plot showing DEPs identified on comparing the samples from two waves and their clustering on PCA.

To better understand the changes in semen proteomics we also compared the samples from recovered COVID-19 patients from the second wave and control individuals, respectively. On comparing the 2nd wave samples with those from control patients, we identified 89 significantly dysregulated proteins, of which 61 were upregulated (increased abundance as compared to control) and 28 were downregulated (decreased abundance as compared to control) (Supplementary Table S3). Of these 89 significant proteins, 14 proteins, voltage-dependent calcium channel subunit alpha-2/delta-1 (CA2D1), glutathione reductase (GSHR), T-complex protein 1 subunit beta (TCPB), Elongation factor 2 (EF2), Fructose-1,6-bisphosphatase 1 (F16P1), heat shock-related 70 kDa protein 2 (HSP72), RAB6B, D-3-phosphoglycerate dehydrogenase (SERA), dystroglycan 1 (DAG1), nucleobindin-1 (NUCB1), SEMG1, cGMP-specific 3′,5′-cyclic phosphodiesterase (PDE5A), Kunitz-type protease inhibitor 2 (SPIT2), and progranulin (GRN) were also identified to be significantly dysregulated when 1^st^ wave samples and controls were compared as reported by Ghosh et al. The Venn diagram illustrates the number of common and unique proteins among the two comparisons and the lollipop plot shows the trends of the common 14 proteins (Figures 2A, B). The volcano plot visualizes the upregulated (61) and downregulated (28) proteins significant in comparing the 2^nd^ wave vs. control samples (Figure 2C). The top 25 significantly dysregulated proteins have been visualized in the form of a heatmap (Supplementary Figure S2A). Finally, we compared the changes in the seminal proteome of the COVID-19 recovered patients infected during the first and second waves. This was to understand how prevalent variants influences the semen parameters. We identified 87 proteins to be significantly dysregulated with 64 upregulated (increased abundance in 2^nd^ wave) and 23 downregulated (decreased abundance in 2^nd^ wave) (Figure 2D, Supplementary Table S4).

**FIGURE 3:**
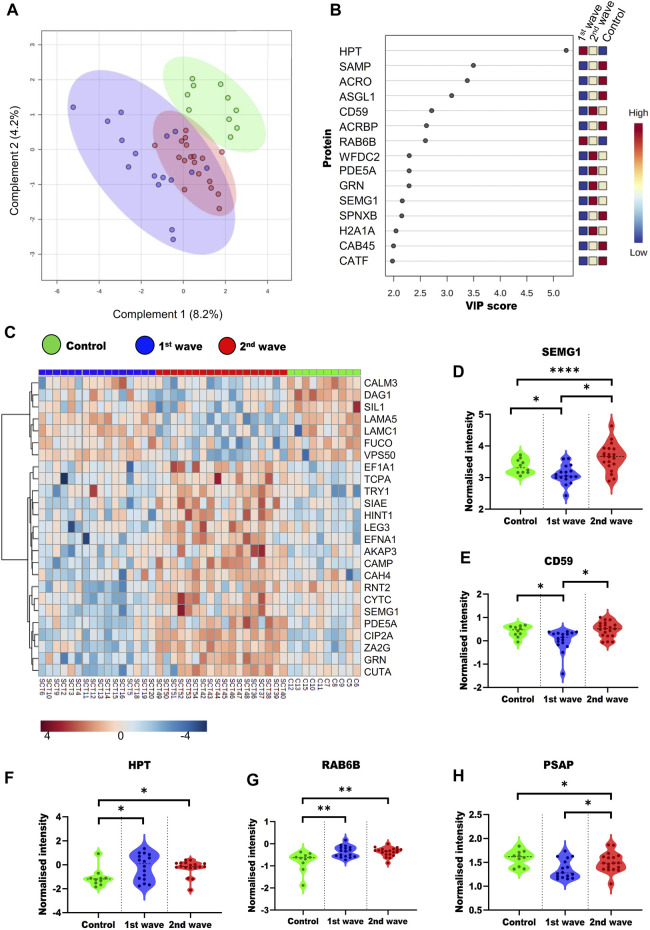
Overall dysregulation in the male reproductive system post recovery from COVID-19 across the different waves **(A)** PLS-DA plot showing the three groups included in study **(B)** Top 15 important proteins based on the VIP scores based on PLS-DA. **(C)** Heatmap visualizing the trends across the three groups for the top 25 proteins based on ANOVA. Violin plots of SEMG1 **(D)**, CD59 **(E)**, HPT **(F)**, RAB6B **(G)**, and PSAP **(H)** proteins show their trend in different groups.

To understand the overall alterations in semen proteome, we compared all the three groups. On PLS-DA, a supervised clustering method, the two groups of COVID-19 recovered patients clustered together, whereas the control group clustered separately (Figure 3A). We also identified the top 15 proteins using VIP scores based on PLS-DA. Figure 3B shows the top 15 proteins their scores and the expression of the protein in a particular group. One way-ANOVA was performed to understand the alteration in the proteome of COVID-19-recovered patients from the 1^st^ and 2^nd^ waves compared to the healthy individuals. Tukey’s HSD was performed as the *post hoc* analysis to identify significantly dysregulated proteins. We identified 69 (Supplementary Table S2) proteins that were significantly dysregulated, of which the top 25 are presented in the form of a heatmap (Figure 3C). Differential abundances of five such proteins, namely, SEMG1, CD59, PSAP, Haptoglobin (HPT), Ras-related protein rab-6b (RAB6B). These proteins have been previously reported as potential biomarker candidates are visualized as violin plots (Figures 3D–H). We also looked at the alterations due to the vaccination status of patients. However, this was done only for second-wave patients. Of these patients, 9 were non-vaccinated and 12 were fully vaccinated. However, we could not identify any significant DEPs based on the vaccination status of the patients.

### 3.2 Pathway analysis

**FIGURE 4:**
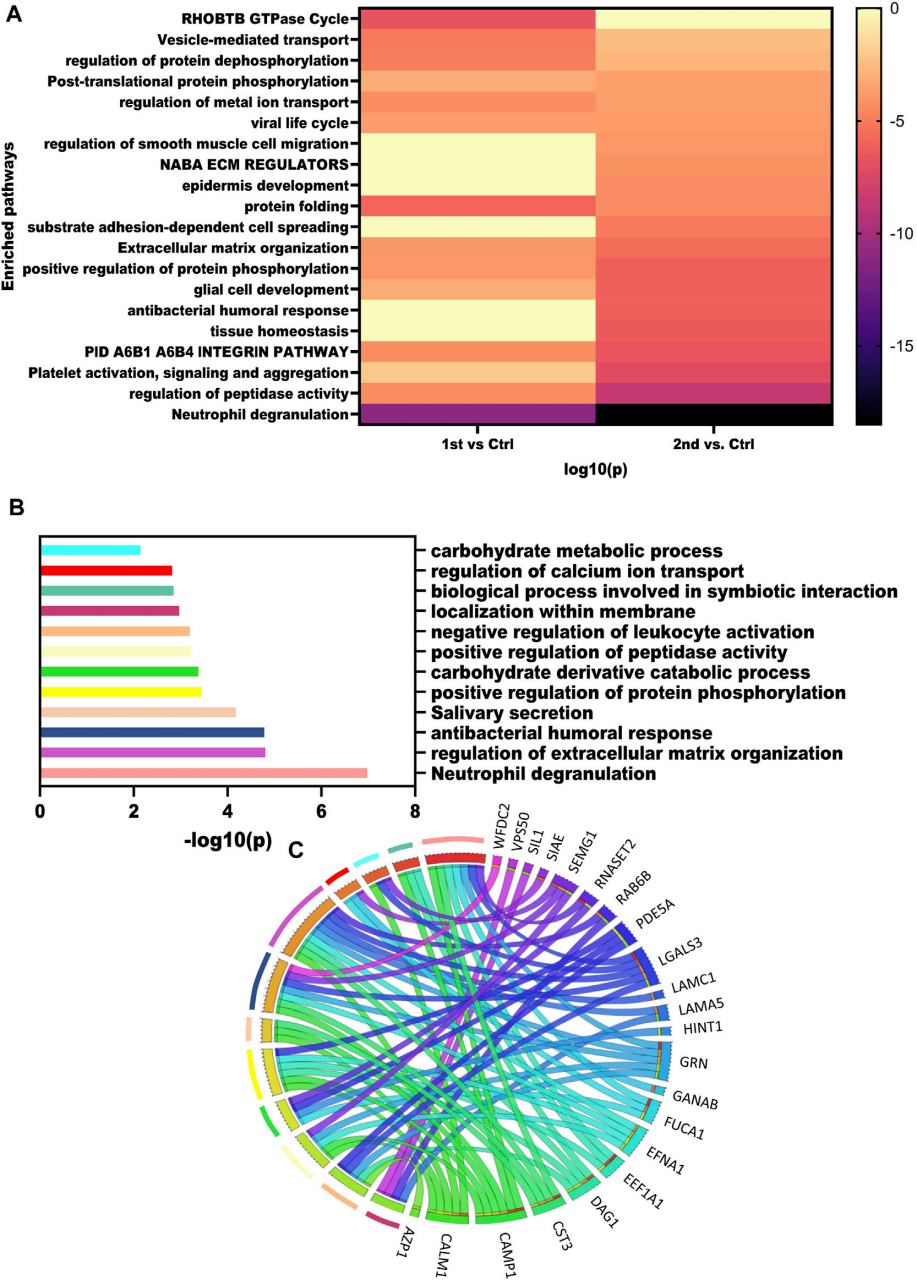
Dysregulated pathways in recovered males. **(A)** Enrichment heatmap showing pathways mapped in different comparisons using all the DEPs identified. **(B)** Bar plot showing the dysregulated pathways based on the top 15 VIP score proteins. **(C)** Circus plot showing the map of VIP proteins and the pathways that were enriched.

To get an overview of pathways dysregulated in these samples we mapped the DEPs obtained from the three comparisons using Metascape. The heatmap illustrates the pathways enriched by the significantly dysregulated proteins from the second wave and first wave (Figure 4A). Neutrophil degranulation was highly enriched by 2^nd^ wave vs. Control comparison. Pathways such as regulation of peptidase activity, extracellular matrix organization, regulation of protein dephosphorylation, regulation of metal ion transport, and vesicle-mediated transport were enriched in all the comparisons. Further, we also performed a pathway analysis using the ANOVA passed proteins. Pathways enriched by ANOVA-passed DEPs of different comparisons have also been listed in Supplementary Table S5. Pathways like spermatogenesis, spermatid development, and spermatid development were also enriched as member pathways under the antibacterial humoral response summary pathway on analysis via Metascape. We also identified the pathways dysregulated by the top 25 proteins identified based on the ANOVA test and visualized using a heatmap. Neutrophil degranulation, regulation of extracellular matrix organization, antibacterial humoral response, Salivary secretion, positive regulation of protein phosphorylation, carbohydrate derivative catabolic process, positive regulation of peptidase activity, negative regulation of leukocyte activation, localization within the membrane, biological process involved in symbiotic interaction, regulation of calcium ion transport, and carbohydrate metabolic process pathways were found to be dysregulated based on the top 25 proteins. The -log10(*p* values) for the pathways were visualized using a bar plot (Figure 4B), whereas the Circos plot has helped us visualize the protein involved in different pathways (Figure 4C).

## 4 Discussion

The pandemic has been challenging the healthcare system with emerging variants. These variants warrant changes in the routine management policies due to the altered infectivity, pathogenicity, and immune escape. In addition, the post-COVID-19 sequelae that transcend beyond the acute pulmonary symptoms also add to the existing burden on the healthcare system. Therefore, understanding the molecular mechanisms underlying the altered infectivity, pathogenesis, and post-COVID-19 sequelae becomes crucial. In addition, there were also vaccine rollouts globally, which was a major step toward controlling the pandemic. India also had the biggest vaccine rollouts by mid-2021 whilst still grappling with the delta-variant-driven COVID-19 resurgence (Juyal et al., 2021). As of May 2021, till the time of sample collection for the second wave, 2.1% of India’s population was fully vaccinated; i.e., they had received two doses of the COVID-19 vaccine (Chakraborty et al., 2021). The delta COVID-19 wave in India, although it lasted for 3 months, was catastrophic. This emphasized the need for increased vaccination and understanding of the effects of vaccination and post-COVID-19 sequelae.

Concerning the male sex, the viral infection was reported to have skewness in the infection rate (Yadav et al., 2021), morbidities, and mortality (Jin et al., 2020). Along with the increase in susceptibility to the virus, there were also reports of changes in the semen parameters post-recovery from COVID-19 (Guo et al., 2021; Tiwari et al., 2021). Semen parameter analysis is the primary method of evaluating male fertility, wherein the macroscopic and microscopic parameters of semen are used to confirm sub/infertility (Agarwal et al., 2016). However, this does not provide insights into alterations at the molecular levels, which could help us better understand the underlying mechanism. Therefore, it warrants an in-depth analysis. Semen comprises both cellular (sperm) and non-cellular components; it provides nourishment and protection to the spermatozoa. Mass-spectrometry-based proteomics approaches can be utilized to determine molecular changes (Samanta et al., 2018). In our earlier study, we presented the first evidence of alterations in semen proteome post-recovery from COVID-19. The study also helped us establish the fact that the male reproductive system is affected post-COVID-19 recovery (Ghosh et al., 2022). Thus, emphasizing the need for overall follow-up of patient’s post-recovery from COVID-19 is maintained. The mRNA-based vaccine has been deemed safe and there was no effect observed on semen parameters (Gonzalez et al., 2021; Lifshitz et al., 2022). Similar findings have also been reported in two studies for non-mRNA-based vaccines such as AstraZeneca. Nonetheless, the molecular changes due to the vaccine remain elusive (Massarotti et al., 2022; Meitei et al., 2022). Moreover, the effect of vaccination on post-COVID-19 sequelae remains elusive. Therefore, we performed an analysis to understand the changes in the male reproductive system in recovered COVID-19 patients across the two waves.

In this study with COVID-19-recovered patients, we identified some male reproductive system-related proteins to be significantly dysregulated. These included highly abundant proteins like SEMG1, SEMG2, and kallikrein 3 (Prostate-specific antigen). However, these proteins have also been reported as biomarkers for infertility. The semenogelins are proteins secreted by the seminal vesicles. These proteins are involved in providing sperm protection from the acidic vaginal environment and form part of the ejaculated coagulum (Lamirande, 2007). Both SEMG1 and SEMG2 have been reported to be downregulated in infertile men. In our samples, SEMG1 and SEMG2 were significantly downregulated in the first wave samples compared to the control. However, in the second wave samples, these proteins were significantly upregulated compared to the 1^st^ wave samples as well as controls. This was observed in another study evaluating the effect of oxidative stress on seminal fluid. They speculated that the increase in the semenogelins level could be due to the incomplete cleavage by KLK3 (PSA) (Lamirande, 2007; Sharma et al., 2013). However, in our study, KLK3 also showed upregulation in the second-wave samples. KLK3 activity is crucial for semen liquification and an increase in KLK3 in seminal plasma has been reported to affect sperm motility in asthenozoospermic (AZ) males. On the other hand, another study showed that increase in SEMG1 in AZ males. However, the exact relationship between the increase in SEMG1 and PSA in AZ males remains to be elucidated. Since there was no sperm abnormality-based comparison made in our study, we cannot make a definitive conclusion whether the increased KLK3 and SEMG1 in AZ males are influencing the overall trends of these proteins.

Apart from these abundant proteins, other proteins involved in an extracellular matrix organization and non-integrin ECM interactions were found to be dysregulated. ADAMTS1 and HTRA1 are ECM proteases that play a role in spermatogenesis (Aydos et al., 2016). APCS has been localized to the mature sperm. However, the implication of its downregulation remains elusive despite its role in tail-associated functions and sperm motility (Malm et al., 2008). It was observed to be downregulated in another proteomic study comparing the proteomic landscape of sub-fertile men with different sperm abnormalities (Becker et al., 2023). The downregulation of these proteins was also observed in both waves, indicating the effect of COVID-19 on spermatogenesis. Whereas, CAP1 is involved in sperm structure and motility. In our study, it was found to be downregulated in the COVID-19-recovered patients. This is also reflected in the clinical semen parameters where sperm average % motility value for 1st and 2nd waves, was lower (1st wave-10%, 2nd wave-30%) as compared to control (50%) (Table S1). The ECM organization pathway was also enriched in other fertility study that compared with changes in secondary infertility with primary fertility (Martins et al., 2020). This indicates that the molecular mechanism underlying infertility remains the same. This paves the way for applying these proteins in a clinical setting for monitoring male reproductive health.

Interestingly, we observed that the significantly dysregulated proteins associated with the second wave enriched the neutrophil degranulation pathway. Immune response or inflammation has been known for its negative impact on male reproductive health. An earlier study reported immune reactions and infections as the most enriched pathways in terms of secondary infertility. Secondary infertility was defined as couples who were able to get pregnant at least once, but not subsequently. The enrichment of immune reactions in the secondary infertility group indicates that immune reactions could induce infertility (Martins et al., 2020). Previous studies have reported that inflammation might affect sperm motility and functionality by inducing oxidative stress (Fraczek and Kurpisz, 2007). The DEPs that mapped to the neutrophil degranulation pathway have been associated with infertility previously. CST3 has been reported to alter sperm motility in infertile patients on chronic hemodialysis. (Silva et al., 2020). The study also emphasized the diagnostic value of this protein to evaluate sub/infertility. SLPI protein is a serine protease that has been associated with sperm function and fertilization. We speculate that the systemic inflammation that persists in symptom-free COVID-19 patients might affect the male reproductive system and cause sub/infertility in recovered men. However, so far, only a study in the mouse model has reported enhanced pathology due to infection with the delta variant. The study saw increased enrichment of inflammation-related proteins in the mouse infected with delta variants than in alpha. In addition, GO enrichment also mapped more immunological pathways than the alpha-challenged mouse (Lee et al., 2022). A similar increase in inflammation-related proteins was observed even in our study. Thus, we can say that inflammatory flare-up that occurs during active infection with the delta variant might persist even after recovery from COVID-19. This emphasizes that post-COVID-19 sequelae are also influenced by the inflammatory responses that persists even after clinical recovery.

## 5 Limitations

The analysis has some limitations and the findings hold within these limitations. First, although the delta variant was highly prevalent during the 2^nd^ wave of COVID-19 in India, we do not have genomic sequencing data to confirm that the participants included in the study were infected by the delta variant. The comparison made between the two waves is based on the assumption that our cohort follows the trend of prevalence observed in the general population. Second, the sample size was small to make a comparison between different sperm abnormalities. Third, the biomarkers should be validated using a larger cohort considering clinical parameters and genome sequencing data for variants. Lastly, whether the markers persistently exhibit the same pattern for all new variants remains elusive as our study only included the patients from alpha and delta-dominant COVID-19 waves. Further, we did not include samples from other disease affecting the male reproductive system and therefore, warrants another study for further validation of the current findings.

## 6 Conclusion

In this study, we have successfully shown that the effect of COVID-19 persists irrespective of the variant and that the vaccination status does not influence post-COVID-19 sequelae in the male reproductive system. The observations made in the study would, therefore, help in strengthening the existing knowledge base about the effect of COVID-19 on the male reproductive system post-recovery. Only looking at the classic biomarker candidates such as SEMG1, KLK3, PSAP, and CAMP would not be sufficient. Therefore, further validation of protein biomarkers associated with sperm function and parameters along with other inflammatory markers based on existing literature using targeted proteomics approaches and a larger cohort is required. The development of protein biomarker panels for monitoring the post-COVID-19 sequelae in the male reproductive system would be crucial in a clinical setting for the early classification of such patients and for devising medical strategies.

## Data Availability

The datasets presented in this study can be found in online repositories. The mass spectrometry-based shotgun proteomics data have been deposited in the ProteomeXchange Consortium via the PRIDE partner repository (https://www.ebi.ac.uk/pride/) with the data set identifier PXD041904.
