# Supplementary figures and images for: Semen proteomics reveals alterations in fertility-related proteins post-recovery from COVID-19
Source: Front Physiol. 2023 Nov 9;14:1212959. doi: 10.3389/fphys.2023.1212959 (PMC10665489; doi:10.3389/fphys.2023.1212959)

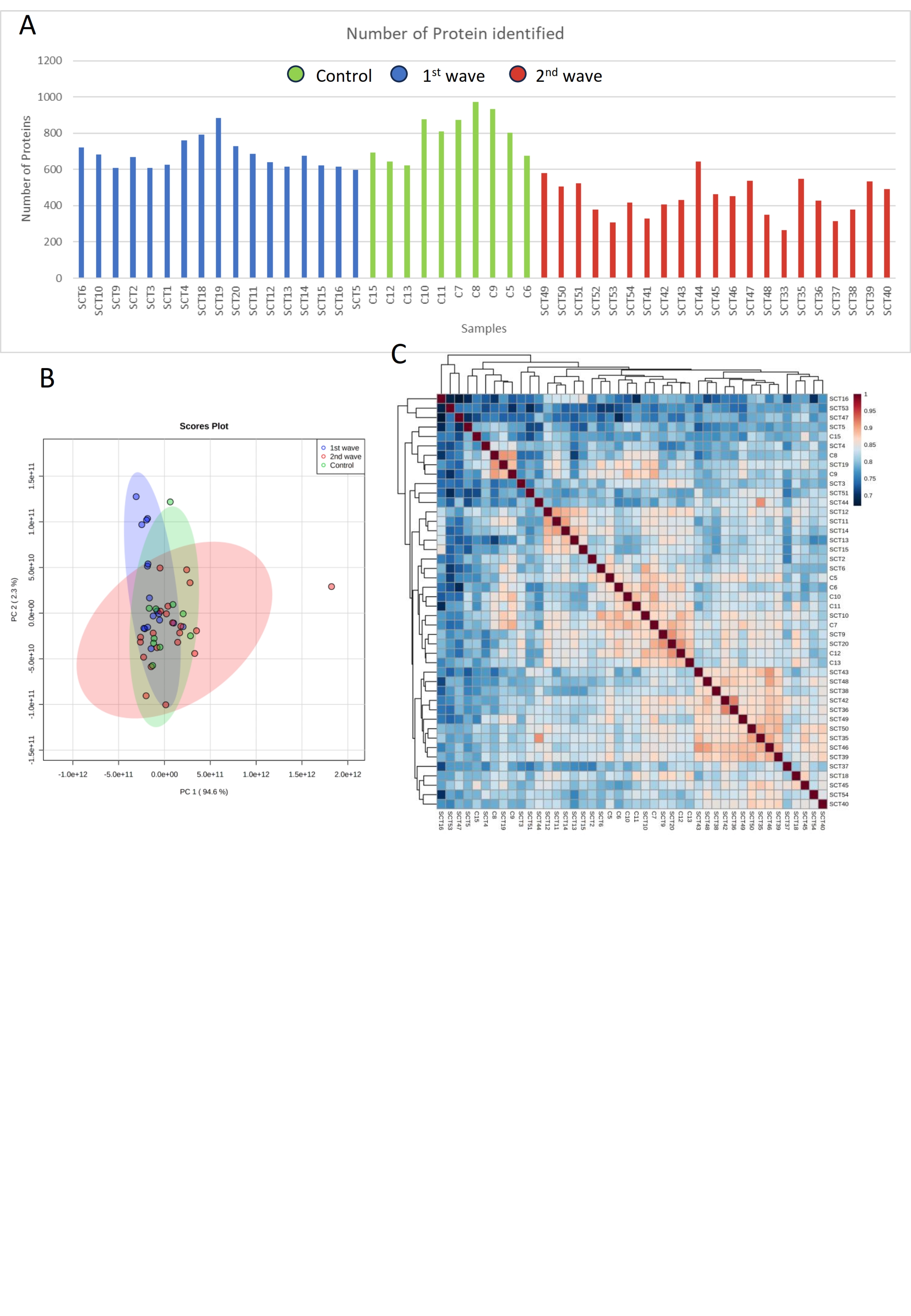

Supplement: Supplementary file 4 [file Image1.JPEG]

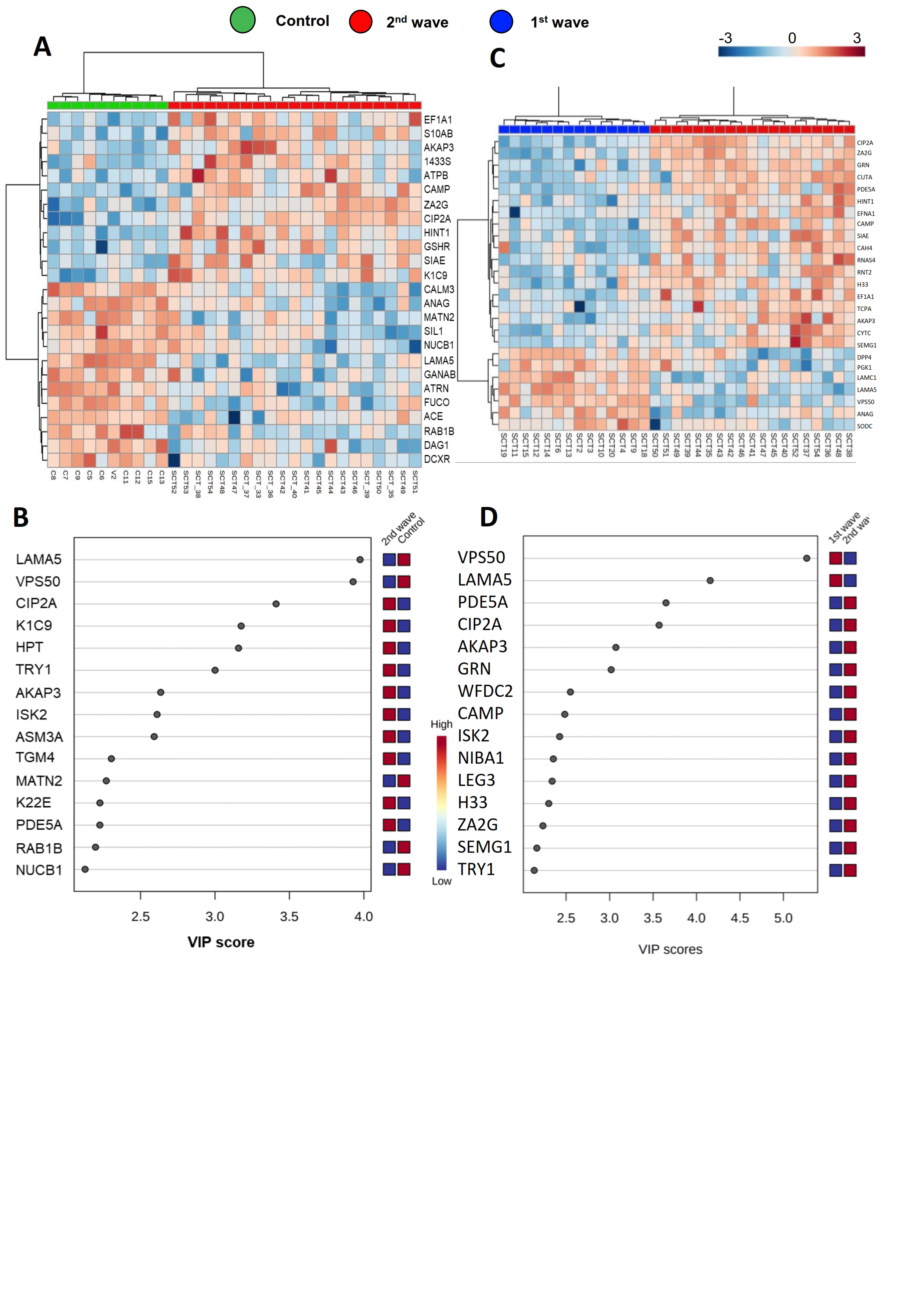

Supplement: Supplementary file 5 [file Image2.JPEG]
