# Supplementary material for: Semen proteomics reveals alterations in fertility-related proteins post-recovery from COVID-19
Source: Front Physiol. 2023 Nov 9;14:1212959. doi: 10.3389/fphys.2023.1212959 (PMC10665489; doi:10.3389/fphys.2023.1212959)
Supplement: Supplementary file 7 [file DataSheet2.DOCX]

Supplementary data sheet


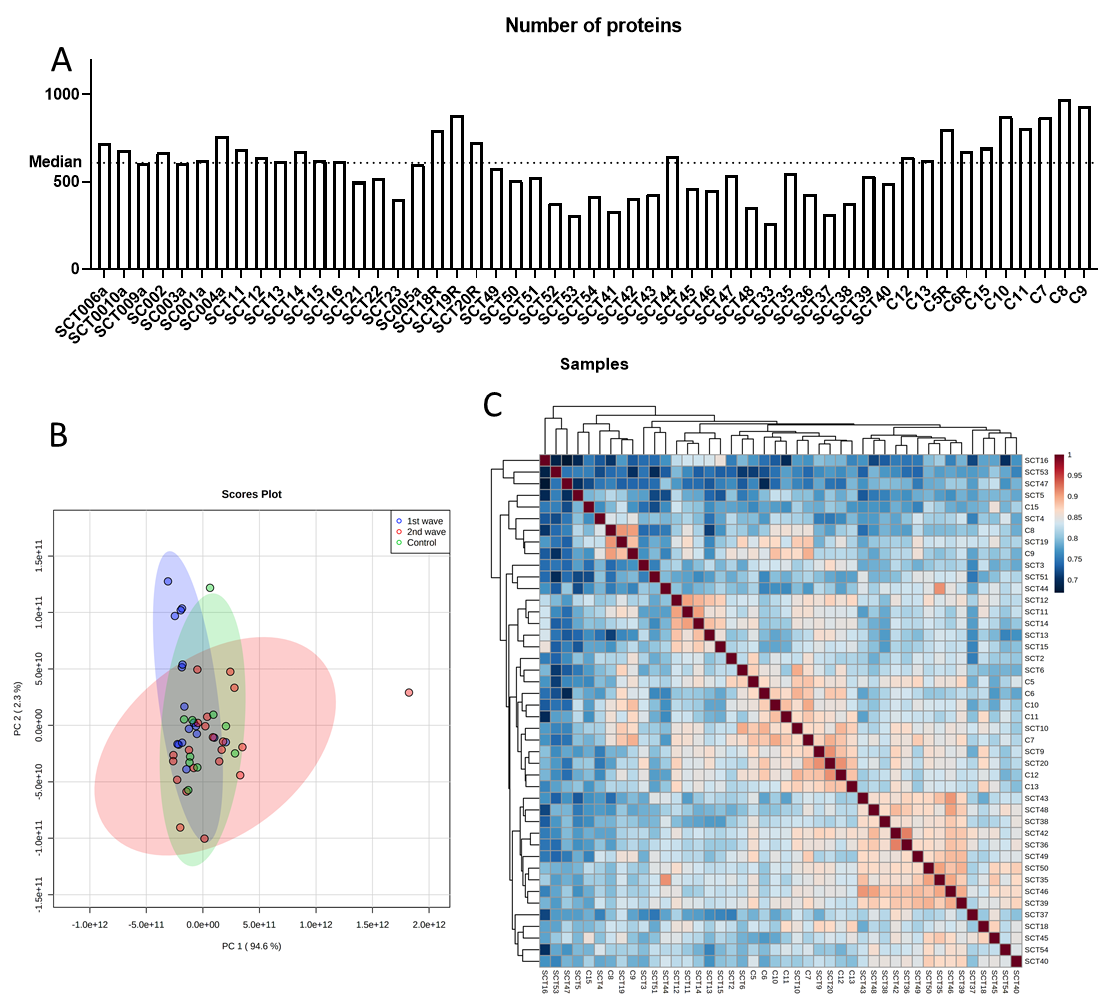


**Supplementary Figure 1.** A) Number of proteins identified for all the samples, B) PCA plot showing clustering of samples-indicating no batch effect, C) Correlation heatmap for all the samples included in the study.


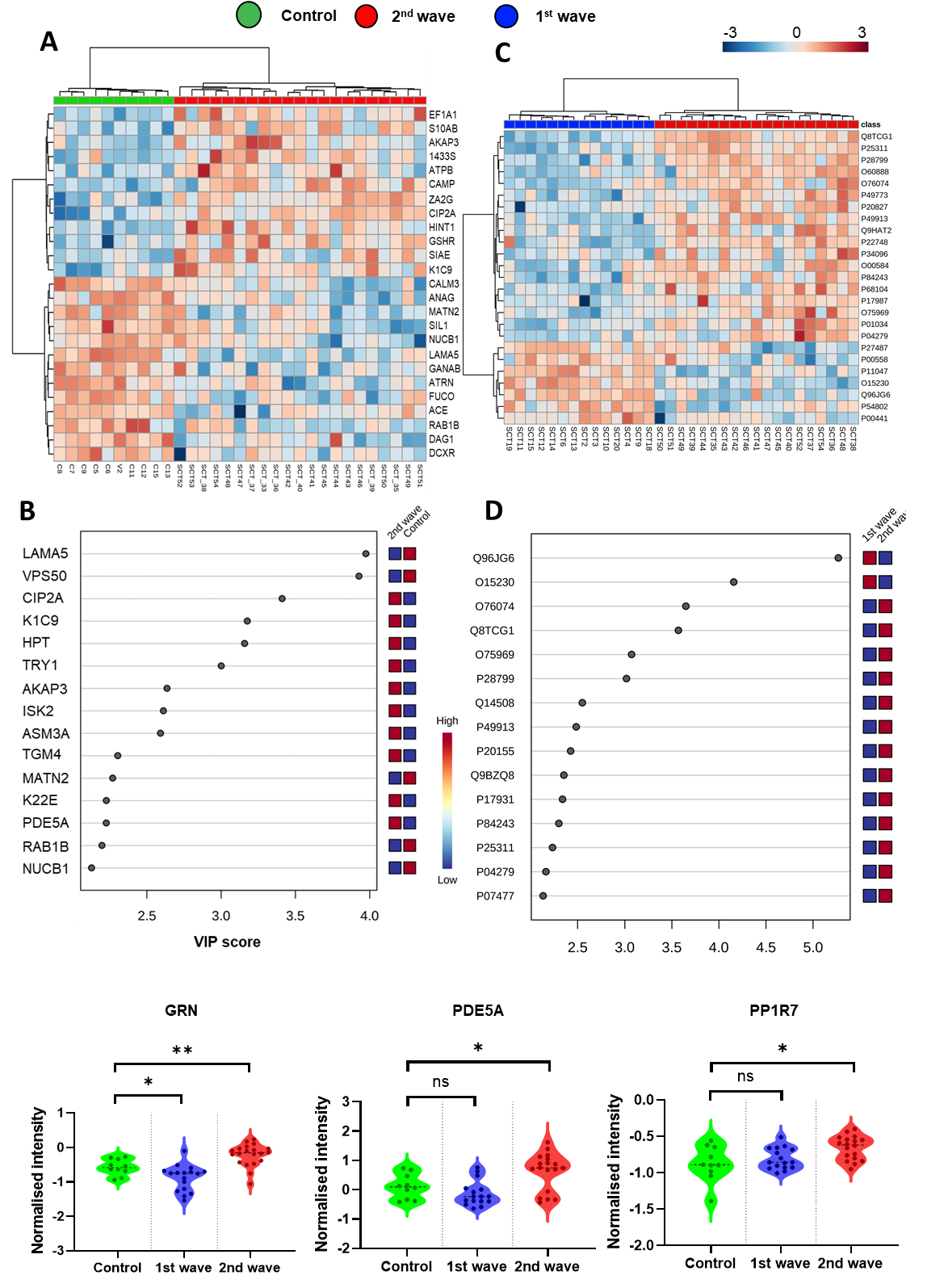


**Supplementary Figure 2**.A) Heatmap visualising trends of top 25 DEPs identified in 2^nd^ wave. B) Top 15 VIP score proteins identified in 2^nd^ waves samples C) Heatmap visualising trends of top 25 DEPs identified on comparing 2^nd^ wave samples with 1^st^ wave. D) Top 15 VIP score proteins identified in on comparing 2^nd^ wave samples with 1^st^ wave
