# Supplementary material for: Semen proteomics reveals alterations in fertility-related proteins post-recovery from COVID-19
Source: Front Physiol. 2023 Nov 9;14:1212959. doi: 10.3389/fphys.2023.1212959 (PMC10665489; doi:10.3389/fphys.2023.1212959)
Supplement: Supplementary file 9 [file DataSheet1.DOCX]

**Supplementary File S1:** Maxquant Parameters

| Parameter | Value |
| --- | --- |
| Version | 1.6.6.0 |
| User name | Admin |
| Machine name | DESKTOP-9UL5R73 |
| Date of writing | 08-04-2022 07:39 |
| Include contaminants | TRUE |
| PSM FDR | 0.01 |
| PSM FDR Crosslink | 0.01 |
| Protein FDR | 0.01 |
| Site FDR | 0.01 |
| Use Normalized Ratios For Occupancy | TRUE |
| Min. peptide Length | 7 |
| Min. score for unmodified peptides | 0 |
| Min. score for modified peptides | 40 |
| Min. delta score for unmodified peptides | 0 |
| Min. delta score for modified peptides | 6 |
| Min. unique peptides | 1 |
| Min. razor peptides | 1 |
| Min. peptides | 1 |
| Use only unmodified peptides and | TRUE |
| Modifications included in protein quantification | Oxidation (M);Acetyl (Protein N-term) |
| Peptides used for protein quantification | Razor |
| Discard unmodified counterpart peptides | TRUE |
| Label min. ratio count | 2 |
| Use delta score | FALSE |
| iBAQ | FALSE |
| iBAQ log fit | FALSE |
| Match between runs | TRUE |
| Matching time window [min] | 0.7 |
| Match ion mobility window [indices] | 0.05 |
| Alignment time window [min] | 10 |
| Alignment ion mobility window [indices] | 1 |
| Find dependent peptides | FALSE |
| Fasta file | F:\Semen Data\2022_as\uniprot-human-filtered-reviewed_yes+AND+organism__Homo+sapiens+(Human)--.fasta |
| Decoy mode | revert |
| Include contaminants | TRUE |
| Advanced ratios | TRUE |
| Fixed andromeda index folder | |
| Temporary folder |  |
| Combined folder location | |
| Second peptides | TRUE |
| Stabilize large LFQ ratios | TRUE |
| Separate LFQ in parameter groups | FALSE |
| Require MS/MS for LFQ comparisons | TRUE |
| Calculate peak properties | FALSE |
| Main search max. combinations | 200 |
| Advanced site intensities | TRUE |
| Write msScans table | FALSE |
| Write msmsScans table | TRUE |
| Write ms3Scans table | TRUE |
| Write allPeptides table | TRUE |
| Write mzRange table | TRUE |
| Write pasefMsmsScans table | TRUE |
| Write accumulatedPasefMsmsScans table | TRUE |
| Max. peptide mass [Da] | 4600 |
| Min. peptide length for unspecific search | 8 |
| Max. peptide length for unspecific search | 25 |
| Razor protein FDR | TRUE |
| Disable MD5 | FALSE |
| Max mods in site table | 3 |
| Match unidentified features | FALSE |
| Epsilon score for mutations | |
| Evaluate variant peptides separately | TRUE |
| Variation mode | None |
| MS/MS tol. (FTMS) | 20 ppm |
| Top MS/MS peaks per Da interval. (FTMS) | 12 |
| Da interval. (FTMS) | 100 |
| MS/MS deisotoping (FTMS) | TRUE |
| MS/MS deisotoping tolerance (FTMS) | 7 |
| MS/MS deisotoping tolerance unit (FTMS) | ppm |
| MS/MS higher charges (FTMS) | TRUE |
| MS/MS water loss (FTMS) | TRUE |
| MS/MS ammonia loss (FTMS) | TRUE |
| MS/MS dependent losses (FTMS) | TRUE |
| MS/MS recalibration (FTMS) | FALSE |
| MS/MS tol. (ITMS) | 0.5 Da |
| Top MS/MS peaks per Da interval. (ITMS) | 8 |
| Da interval. (ITMS) | 100 |
| MS/MS deisotoping (ITMS) | FALSE |
| MS/MS deisotoping tolerance (ITMS) | 0.15 |
| MS/MS deisotoping tolerance unit (ITMS) | Da |
| MS/MS higher charges (ITMS) | TRUE |
| MS/MS water loss (ITMS) | TRUE |
| MS/MS ammonia loss (ITMS) | TRUE |
| MS/MS dependent losses (ITMS) | TRUE |
| MS/MS recalibration (ITMS) | FALSE |
| MS/MS tol. (TOF) | 40 ppm |
| Top MS/MS peaks per Da interval. (TOF) | 10 |
| Da interval. (TOF) | 100 |
| MS/MS deisotoping (TOF) | TRUE |
| MS/MS deisotoping tolerance (TOF) | 0.01 |
| MS/MS deisotoping tolerance unit (TOF) | Da |
| MS/MS higher charges (TOF) | TRUE |
| MS/MS water loss (TOF) | TRUE |
| MS/MS ammonia loss (TOF) | TRUE |
| MS/MS dependent losses (TOF) | TRUE |
| MS/MS recalibration (TOF) | FALSE |
| MS/MS tol. (Unknown) | 0.5 Da |
| Top MS/MS peaks per Da interval. (Unknown) | 8 |
| Da interval. (Unknown) | 100 |
| MS/MS deisotoping (Unknown) | FALSE |
| MS/MS deisotoping tolerance (Unknown) | 0.15 |
| MS/MS deisotoping tolerance unit (Unknown) | Da |
| MS/MS higher charges (Unknown) | TRUE |
| MS/MS water loss (Unknown) | TRUE |
| MS/MS ammonia loss (Unknown) | TRUE |
| MS/MS dependent losses (Unknown) | TRUE |
| MS/MS recalibration (Unknown) | FALSE |
| Site tables | Oxidation (M)Sites.txt |
